# Supplementary material for: Meta-analysis reveals glucocorticoid levels reflect variation in metabolic rate, not ‘stress’
Source: eLife. 2023 Oct 27;12:RP88205. doi: 10.7554/eLife.88205 (PMC10611431; doi:10.7554/eLife.88205)
Supplement: Supplementary file 1. [file elife-88205-supp1.docx]

| **“energy expenditure” AND (glucocorticoid OR cortisol OR corticosterone) (20/07/2021)** | | | | |
| --- | --- | --- | --- | --- |
| **Total** | **After abstract** | **After text** | **After effect size calculations** |  |
| 707 | 108 | 43 | 9 |  |
| **“metabolic rate” AND (glucocorticoid OR cortisol OR corticosterone) (20/07/2021)** | | | | |
| **Total** | **After abstract** | **After text** | **After effect size calculations** |  |
| 447 | 71 (64*) | 28 | 5 |  |
| **Selected papers’ reference check** | | | | |
| **Total**  **(excl. previously included)** | **After abstract** | **After text** | **After effect size calculations** |  |
| 20 | 15 | 9 | 7 |  |
| *Text step requires paper selection criteria to be met. Effect size calculation step requires enough data reported to calculate effect sizes by experimental treatment.*  *Main reasons for not including articles after abstract / text step: i) Experimental treatment had no effect on metabolic rate, ii) chemical or drug induction of metabolic rate increase, iii) GCs were measured in other tissues than plasma, iv) no experimental manipulation of metabolic rate.*  **Number of studies excluding those overlapping with previous search, which were not further considered.* | | | |  |
