# Supplementary material for: Meta-analysis reveals glucocorticoid levels reflect variation in metabolic rate, not ‘stress’
Source: eLife. 2023 Oct 27;12:RP88205. doi: 10.7554/eLife.88205 (PMC10611431; doi:10.7554/eLife.88205)
Supplement: Supplementary file 4. [file elife-88205-supp4.docx]

| **(a)** | **Estimate** | **s.e.** | **Z** | **P** | **95% C.I.** |
| --- | --- | --- | --- | --- | --- |
| Intercept | 0.70 | 0.12 | 6.00 | <0.0001 | 0.47-0.93 |
| **MR effect size (ln)** | **0.29** | **0.11** | **2.70** | **0.007** | **0.08-0.50** |
| Taxa (mammal) | 0.12 | 0.24 | 0.49 | 0.63 | -0.35-0.58 |
| MR : Taxa | 0.17 | 0.22 | 0.80 | 0.42 | -0.25-0.60 |
| Variance components: Study.ID (Sigma^2)– Estimate = 0.00, sqrt = 0.00, n= 21  Residual heterogeneity: QE(df = 31) = 27.44, p = 0.65  Test of moderators: QM(df = 3) = 9.60, p= 0.022 | | | | | |
| **(b)** | **Estimate** | **s.e.** | **Z** | **P** | **95% C.I.** |
| Intercept | 0.73 | 0.11 | 6.42 | <0.0001 | 0.51-0.95 |
| **MR effect size (ln)** | **0.31** | **0.11** | **2.95** | **0.003** | **0.10-0.52** |
| Before / after effect (yes) | -0.26 | 0.38 | -0.70 | 0.49 | -0.99-0.48 |
| MR : Time effect | 0.02 | 0.31 | 0.05 | 0.96 | -0.59-0.62 |
| Variance components: Study.ID (Sigma^2)– Estimate = 0.00, sqrt = 0.00, n= 21  Residual heterogeneity: QE(df = 31) = 27.91, p = 0.62  Test of moderators: QM(df = 3) = 9.13, p= 0.028 | | | | | |
| **(c)** | **Estimate** | **s.e.** | **Z** | **P** | **95% C.I.** |
| Intercept | 0.73 | 0.11 | 6.51 | <0.0001 | 0.51-0.96 |
| **MR effect size (ln)** | **0.31** | **0.10** | **2.97** | **0.003** | **0.11-0.51** |
| Exp./ control effect (yes) | 0.05 | 0.31 | 0.16 | 0.87 | -0.56-0.65 |
| MR : Exp. / control effect | -0.06 | 0.30 | -0.22 | 0.83 | -0.64-0.51 |
| Variance components: Study.ID (Sigma^2)– Estimate = 0.00, sqrt = 0.00, n= 21  Residual heterogeneity: QE(df = 31) = 28.18, p = 0.61  Test of moderators: QM(df = 3) = 8.94, p= 0.03 | | | | | |
| **(d)** | **Estimate** | **s.e.** | **Z** | **P** | **95% C.I.** |
| Intercept | 0.73 | 0.12 | 6.11 | <0.0001 | 0.50-0.97 |
| **MR effect size (ln)** | **0.33** | **0.11** | **2.97** | **0.003** | **0.11-0.54** |
| Met. variable (HR) | 0.14 | 0.24 | 0.57 | 0.57 | -0.33-0.60 |
| MR : Met. variable | -0.02 | 0.22 | -0.07 | 0.94 | -0.45-0.42 |
| Variance components: Study.ID (Sigma^2)– Estimate = 0.01, sqrt = 0.09, n= 21  Residual heterogeneity: QE(df = 31) = 28.09, p = 0.62  Test of moderators: QM(df = 3) = 8.67, p= 0.022 | | | | | |
| **e)** | **Estimate** | **s.e.** | **Z** | **P** | **95% C.I.** |
| Intercept | 0.78 | 0.21 | 3.79 | 0.0002 | 0.38-1.19 |
| **MR effect size (ln)** | **0.27** | **0.19** | **1.39** | **0.166** | **-0.11-0.64** |
| Treat. Type 2 | -0.03 | 0.27 | -0.09 | 0.93 | -0.56-0.51 |
| Treat. Type 3 | -0.16 | 0.31 | -0.52 | 0.60 | -0.78-0.45 |
| MR : Treat. Type 2 | 0.05 | 0.25 | 0.22 | 0.83 | -0.44-0.55 |
| MR : Treat. Type 3 | 0.08 | 0.29 | 0.27 | 0.79 | -0.48-0.64 |
| Variance components: Study.ID (Sigma^2)– Estimate = 0.02, sqrt = 0.14, n= 21  Residual heterogeneity: QE(df = 29) = 27.99, p = 0.52  Test of moderators: QM(df = 5) = 8.86, p= 0.115 | | | | | |
